# Dutch ICU survivors have more consultations with general practitioners before and after ICU admission compared to a matched control group from the general population

**DOI:** 10.1371/journal.pone.0217225

**Published:** 2019-05-23

**Authors:** Ilse van Beusekom, Ferishta Bakhshi-Raiez, Nicolette F. de Keizer, Marike van der Schaaf, Fabian Termorshuizen, Dave A. Dongelmans

**Affiliations:** 1 Amsterdam UMC, University of Amsterdam, Department of Medical Informatics, Amsterdam Public Health Research Institute, Amsterdam, The Netherlands; 2 National Intensive Care Evaluation (NICE) foundation, Amsterdam, The Netherlands; 3 Amsterdam UMC, University of Amsterdam, Department of Rehabilitation, Amsterdam Movement Sciences, Amsterdam, The Netherlands; 4 Centre of Applied Research, Faculty of Health, Amsterdam University of Applied Sciences, Amsterdam, The Netherlands; 5 Amsterdam UMC, University of Amsterdam, Department of Intensive Care Medicine, Amsterdam, The Netherlands; University of Notre Dame Australia, AUSTRALIA

## Abstract

**Background:**

General Practitioners (GPs) play a key role in the healthcare trajectory of patients. If the patient experiences problems that are typically non-life-threatening, such as the symptoms of post-intensive-care syndrome, the GP will be the first healthcare professional they consult. The primary aim of this study is to gain insight in the frequency of GP consultations during the year before hospital admission and the year after discharge for ICU survivors and a matched control group from the general population. The secondary aim of this study is to gain insight into differences between subgroups of the ICU population with respect to the frequency of GP consultations.

**Methods:**

We conducted a retrospective cohort study, combining a national health insurance claims database and a national quality registry for ICUs. Clinical data of patients admitted to an ICU in 2013 were enriched with claims data from the years 2012, 2013 and 2014. Poisson regression was used to assess the differences in frequency of GP consultations between the ICU population and the control group.

**Results:**

ICU patients have more consultations with GPs during the year before and after admission than individuals in the control group. In the last four weeks before admission, ICU patients have 3.58 (CI 3.37; 3.80) times more GP consultations than the control group, and during the first four weeks after discharge they have 4.98 (CI 4.74; 5.23) times more GP consultations. In the year after hospital discharge ICU survivors have an increased GP consultation rate compared to the year before their hospital admission.

**Conclusions:**

Close to hospital admission and shortly after hospital discharge, the frequency of GP consultations substantially increases in the population of ICU survivors. Even a year after hospital discharge, ICU survivors have increased GP consultation rates. Therefore, GPs should be well informed about the problems ICU patients suffer after discharge, in order to provide suitable follow-up care.

## Introduction

ICU survivors suffer long-term and severe complaints such as physical, mental, and cognitive impairments, limitations in daily and social activities, and problems affecting their work and employment, [1, 2] all leading to a reduced quality of life. The term ‘Post Intensive Care Syndrome’ (PICS) was introduced to describe the presence of one or more physical, cognitive or mental impairments after critical illness [3]. Although the exact prevalence of PICS among ICU survivors is unknown, it is estimated that 25–50% of ICU survivors will suffer from some component of PICS after hospital discharge [4–6].

ICU follow-up care has been suggested as a means to address the problems faced after discharge, but it is unknown which (combination of) interventions are most (cost)effective [7, 8]. There is currently insufficient awareness with regards to PICS among clinicians, survivors, families, healthcare administrators, and policymakers, resulting in insufficient treatment of the complete scope of PICS [3, 9, 10]. Moreover, there is evidence that half of the ICU survivors with complaints had no contact with the appropriate health professional at three months after hospital discharge [10].

As in many Northwestern European countries, in the Dutch healthcare system the general practitioner (GP) plays a key role in the healthcare trajectory of all patients and acts as a gatekeeper between the patient and other healthcare providers. If the patient experiences problems that are typically non-life-threatening, the GP will be the first healthcare professional they consult. If needed, the GP refers the patients to the right healthcare provider. This raises the question of whether this is also the case when ICU survivors consult their GP about complaints experienced as part of PICS.

A first step is to gain insight in the current situation. To date, it is unknown whether ICU survivors contact their GP more often compared to the general population, and if this changes over time. In addition, little is known about differences between ICU subgroups with respect to number of GP consultations. This knowledge could be of great importance for GPs, policy makers, intensivists, healthcare insurers and care planners in order to get insight into the GP’s potential role in organising care tailored to the needs of ICU survivors. Therefore, the primary aim of this study is to gain insight in the frequency of GP consultations during the year before hospital admission and the year after hospital discharge, and investigate trends in time for ICU survivors, compared to people who have not been admitted to an ICU. The secondary aim of this study is to gain insight in the frequency of GP consultations within subgroups of ICU patients.

## Methods

For this project, we combined data from the Dutch National Intensive Care Evaluation (NICE) registry [11] with data from the health insurance claims database of Vektis [12] and conducted a retrospective cohort study.

### Databases

#### Dutch national intensive care evaluation database

The NICE registry is a national quality registry and during the study period 90% of all Dutch ICUs were participating [11]. All participating ICUs collect demographic, clinical, and physiological data for all patients admitted to their ICU. The registry includes among others: age, gender, ICU admission and discharge data, primary diagnosis at ICU admission, ICU mortality, in-hospital mortality and all variables required to quantify the severity of illness and to calculate case-mix adjusted mortality risks according to the Acute Physiology and Chronic Health Evaluation (APACHE) IV model [13]. All patients from the NICE registry, aged 18 years or older during the year of ICU admission, admitted to an ICU during the year 2013 and discharged from the ICU before January 1st 2014, were included in the NICE registry subset for this study.

#### Vektis insurance claims database

Healthcare insurance is compulsory for all Dutch residents and essentially all (99%) of the Dutch inhabitants have private healthcare insurance [14]. The Vektis databases [12] contain reimbursement data on all medical treatments paid for by Dutch insurance companies, as well as demographic information, such as gender, date of birth and a proxy for date of death.

Vektis includes all claims of GPs in the GP Information System. This information system contains information about all claims for GP consultations (face-to-face, telephone, mail) and all medical examinations and tests performed by the GP. Claims for consultations with nurse practitioners, working under the responsibility of the GP, are present in the dataset as well, along with claims for the capitation fees. For this study all claims for consultations, medical examinations and tests are included as well as all consultations with nurse practitioners.

For the treatment and supervision of specified chronic conditions (DM type II, cardiovascular risk management, COPD) the GP can, if desired, make arrangements with healthcare insurance companies. For people with ‘multidisciplinary care arrangements’, there is a fixed price for all care from the GP for the treatment of the specific chronic condition. This is why separate consultations for the specified conditions are not registered. All other consultations of these patients that have no relation to the specified chronic condition are registered. Therefore we can only include the GP consultations with no relation to the specified chronic condition in the analyses for people with multidisciplinary care arrangements.

Vektis also contains claims for pharmaceutical care, including information on provided drugs, the Anatomical Therapeutic Chemical (ATC) code, the date the drug was supplied, and the quantity supplied. To determine the chronic conditions, Pharmaceutical Cost Groups (PCGs) were used as a proxy. PCGs are based on the idea that a patient with a certain chronic condition can be identified by claims for specific prescribed drugs [15, 16]. We used the PCGs to identify chronic conditions during the whole study period since clinical diagnosis are not available within the NICE registry or the Vektis databases. A complete description of the definitions of chronic conditions and ATC codes is published before [17].

The socio-economic status (SES) was derived from the postal code of a person and the SES score for that postal code as determined by the Netherlands Institute for Social Research [18]. The SES score is based on the mean income of a code where a person lives, the fraction of people with a low income, the fraction of people with low education and the fraction of unemployed people. The SES score is ranked and the national mean is 0 (range −6.65; 3.02). A lower score indicates a lower SES and a higher scores indicates a higher SES.

All patients in the Vektis database who were 18 years or older and had a claim for an ICU day in the year 2013 were included in the ICU-subset of the Vektis database. Based on this ICU-subset, a population based control group was created from all registered inhabitants of the Netherlands in the Vektis database. The population based control group was frequency matched based on the combination of the age, gender, and SES of patients from the ICU-subset from the Vektis database, and had no claims for ICU care during 2013. Only ICU patients with no missing data for gender, age and SES were used in the frequency matching process. The frequency matching process was undertaken before the linking process.

### Linking and 1:1 matching process

The ICU-subset extracted from the Vektis database and the NICE database were linked using a deterministic linkage algorithm [19]. A detailed description of the linking process is published previously [20]. Before the 1:1 matching process, ICU patients who did not survive their hospital admission were excluded, as these patients have no GP consultations during the year after discharge. The remaining ICU patients were matched 1:1 with control persons. The 1:1 matching was performed on age, gender and quartile of SES.

### Outcome measures

The primary outcome of this study is the difference in GP consultation rate between the ICU population and the control group during the year before hospital admission and the year after hospital discharge. Based on the hospital admission date of the ICU patient, all contacts with the GP during the year before hospital admission were identified. The hospital discharge date associated with the last ICU admission during 2013 was used to identify all contacts with the GP during the year after hospital discharge. For the control patients, the hospital admission date and the hospital discharge date of their 1:1 matched ICU patient were used to calculate the year before admission and the year after discharge.

### Statistical analysis

Descriptive statistics were used to characterize demographic data of both study populations. Medians and IQR are provided for continuous data and numbers and proportions are used to present categorical data. The Chi-square test was used to test for differences in proportions between the ICU population and control group.

The mean number of GP consultations per week is calculated to gain insight in the trend over time. The difference in number of GP consultations between the ICU population and the control group, expressed as a Risk Ratio, was estimated using Poisson regression. Overdispersion was taken into account by adding a scale parameter to the estimated variance parameter and time at risk was taken into account by adding this as an offset to the regression model. Age, gender, quartiles of SES and number of chronic conditions were considered as confounders and as possible effect modifiers. Age was stratified into the following subgroups: < = 29, 30–39, 40–49, …, 70–79, 80–89, > = 90 and number of chronic conditions was categorized as zero chronic conditions, one chronic condition, two chronic conditions and more than two chronic conditions.

Analyses were performed for the total study period, the year before hospital admission and the year after hospital discharge. Furthermore, the year before admission and the year after discharge were each divided into three timeframes based on the discontinuity of patterns in mean number of GP consultations.

For the secondary aim, the ICU population was divided into subgroups based on the type of ICU admission (medical admission, emergency surgery or elective surgery), the length of ICU stay categorized as <2 days, 2 days to 5 days and > = 5 days, and for the APACHE IV predicted mortality [13] categorized as low-risk (predicted mortality <30%), medium-risk (predicted mortality 30%-70%) and high-risk (predicted mortality ≥70%). We performed sub-analyses for these subgroups and age, gender and quartiles of SES were taken into account as confounders.

All statistical analyses were performed in SAS software (version 7.1; SAS Institute Inc, Cary, NC) and a *p*-value of <0.05 was considered to indicate a statistically significant difference.

## Results

**Fig 1:**
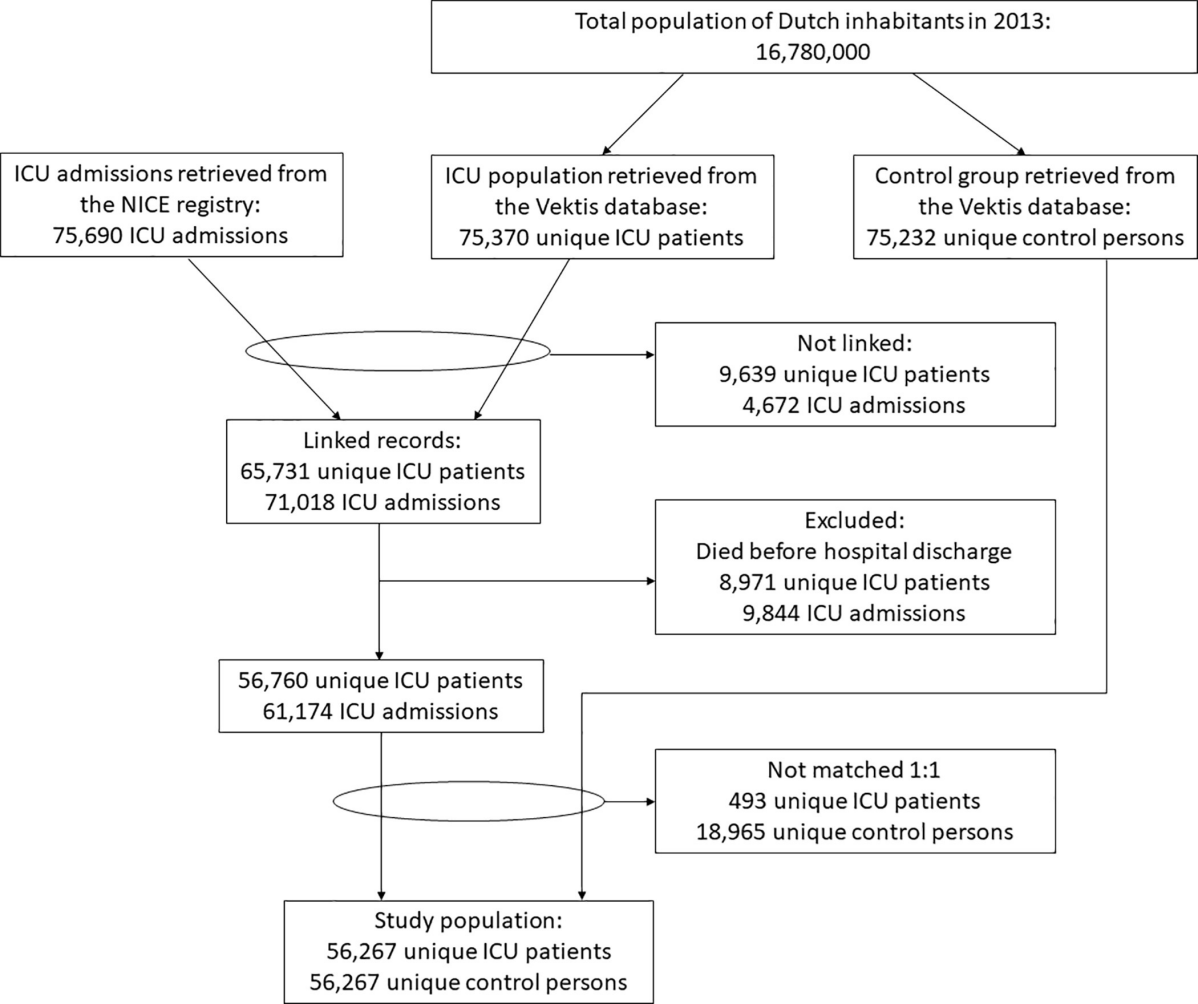
Flowchart of the linking and matching process.

The final study population consisted of 56,267 ICU patients and an equal number of matched persons in the population-based control group. An overview of the data linkage and data matching process is given in Fig 1. Demographic information of the ICU patients and the control group is given in Table 1.

**Table 1:** Demographic information of the ICU population and the control group.

	ICU population (n = 56,267)	Control group (n = 56,267)
Gender (male)	33,825 (60.1%)	33,825 (60.1%)
Age	65 (54; 73)	65 (54; 73)
SES	0.17 (-0.60; 0.79)	0.17 (-0.60; 0.79)
Mortality during study period (2012–2014)	5,923 (10.5%)	1,644 (2.9%)
Population with > = 1 chronic conditions	31,278 (55.6%)	21,187 (37.7%)
Population with > = 2 chronic conditions	10,799 (19.2%)	5,012 (8.9%)
Characteristics of the first ICU admission	
Admission type	
• Medical	22,527 (40.0%)
• Planned surgery	26,714 (47.5%)
• Emergency surgery	6,844 (12.2%)
Length of ICU stay in days	1.0 (0.8; 2.5)
Length of hospital stay in days	9.0 (5.6; 16.0)
APACHE IV score*[13]	49 (36; 65)

* Only calculated for ICU admissions which met the APACHE IV inclusion criteria (n = 53,737 (95.5%)) [13]

APACHE IV: Acute Physiology and Chronic Health Evaluation IV; ICU: Intensive Care Unit; SES: Socioeconomic status

During the year before hospital admission, 3.9% of ICU patients had no contact with a GP. Within the control group this was 16.8% (p<0.0001). During the year after hospital admission, 5.2% of the ICU population and 17.9% of the control group had no contact with the GP (p<0.0001).

The number of ICU patients with multidisciplinary care arrangements during the year before hospital admission was 12,820 (22.8%) and within the control group this was 9,927 (17.6%) (p<0.0001). During the year after hospital discharge 13,083 (23.3%) ICU patients had multidisciplinary care arrangements and 10,860 (19.5%) individuals of the control group (p<0.0001).

**Fig 2:**
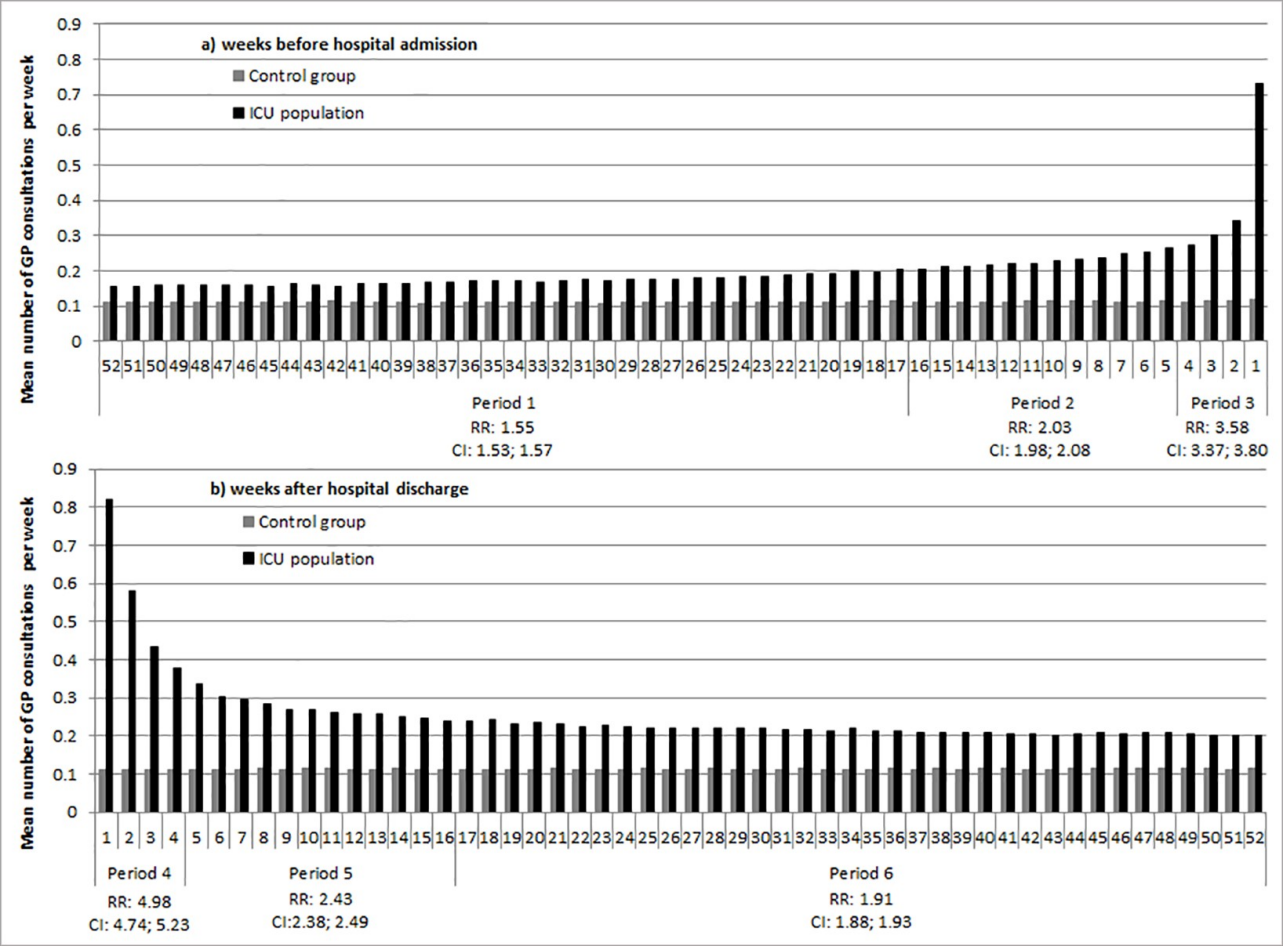
Mean number of GP contacts per week and the crude RR of GP consultations among ICU patients compared to the control group for the different time periods.

Based on the discontinuity of patterns in mean number of GP consultations, the year before admission (Fig 2A) and the year after discharge (Fig 2B) were each divided into three timeframes: 4 weeks before hospital admission/after hospital discharge (period 1 and 4, respectively), 4 weeks to 17 weeks before hospital admission/after hospital discharge (period 2 and 5, respectively) and > = 17 weeks before hospital admission/after hospital discharge (period 3 and 6, respectively).

During the year before hospital admission, ICU patients had 1.82 (CI 1.80; 1.85) times more GP consultations compared to the control group. During the year after hospital discharge, the RR was 2.28 (CI 2.24; 2.31) (Fig 2 and S1 Table).

After adjustment for age, SES and number of chronic conditions, males in the ICU population had 1.36 (CI 1.34; 1.37) times more GP consultations during period 1 compared to males in the control group (Table 2). The RR was 3.46 (CI 3.40; 3.53) during period 3. During period 6, the RR was still increased compared to the same period before hospital admission (RR 1.78 (CI 1.76; 1.79) Table 2). For women within the ICU population as opposed to women of the control group a similar trend was observed.

**Table 2:** Risk Ratios (95% CI) of GP consultations among ICU patients comparted to the control group for different subgroups.

	Year before hospital admission			Year after hospital discharge		
	ICU population Period 1 (week 52–17)	ICU population Period 2 (week 16–5)	ICU population Period 3 (weeks 4–1)	ICU population Period 4 (week 1–4)	ICU population Period 5 (week 5–16)	ICU population Period 6 (week 17–52)
Interaction population*gender ^‡^	*p* = 0.0022	*p* < 0.0001	*p* < 0.0001	*p* < 0.0001	*p* < 0.0001	*p* = 0.0596
Male	1.36 (1.34; 1.37)	1.90 (1.87; 1.92)	3.46 (3.40; 3.53)	4.89 (4.81; 4.97)	2.29 (2.27; 2.32)	1.78 (1.76; 1.79)
Female	1.38 (1.37; 1.40)	1.79 (1.77; 1.81)	3.17 (3.11; 3.24)	3.98 (3.91; 4.06)	2.11 (2.09; 2.14)	1.76 (1.74; 1.77)
Interaction population*SES ^¥^	*p* = 0.0471	*p* < 0.0001	*p* = 0.0009	*p* < 0.0001	*p* < 0.0001	*p* < 0.0001
SES Q1	1.35 (1.33; 1.37)	1.77 (1.74; 1.80)	3.17 (3.09; 3.26)	4.04 (3.94; 4.14)	2.10 (2.07; 2.14)	1.70 (1.68; 1.72)
SES Q2	1.38 (1.37; 1.40)	1.86 (1.83; 1.89)	3.34 (3.24; 3.43)	4.48 (4.37; 4.59)	2.21 (2.17; 2.25)	1.77 (1.75; 1.80)
SES Q3	1.37 (1.35; 1.39)	1.85 (1.82; 1.88)	3.38 (3.28; 3.47)	4.63 (4.52; 4.75)	2.25 (2.21; 2.28)	1.80 (1.77; 1.82)
SES Q4	1.38 (1.36; 1.39)	1.91 (1.88; 1.95)	3.42 (3.33; 3.52)	4.75 (4.64; 4.87)	2.28 (2.24; 2.32)	1.80 (1.78; 1.83)
Interaction population*age ^†^	*p* < 0.0001	*p* < 0.0001	*p* < 0.0001	*p* < 0.0001	*p* < 0.0001	*p* < 0.0001
Age 18–29	1.86 (1.79; 1.94)	2.14 (2.03; 2.26)	3.93 (3.61; 4.28)	4.97 (4.62; 5.36)	2.30 (2.18; 2.42)	2.01 (1.93; 2.09)
Age 30–39	1.84 (1.77; 1.91)	2.28 (2.17; 2.40)	4.22 (3.90; 4.57)	5.52 (5.16; 5.91)	2.52 (2.40; 2.65)	2.08 (2.01; 2.16)
Age 40–49	1.77 (1.73; 1.82)	2.28 (2.21; 2.36)	4.48 (4.26; 4.71)	5.69 (5.44; 5.95)	2.66 (2.57; 2.74)	2.16 (2.10; 2.21
Age 50–59	1.63 (1.60; 1.66)	2.14 (2.09; 2.19)	3.90 (3.76; 4.05)	5.37 (5.20; 5.54)	2.55 (2.49; 2.61)	2.12 (2.08; 2.16)
Age 60–69	1.41 (1.39; 1.43)	1.99 (1.96; 2.03)	3.57 (3.47; 3.67)	5.03 (4.91; 5.14)	2.35 (2.31; 2.39)	1.86 (1.84; 1.89)
Age 70–79	1.25 (1.23; 1.26)	1.73 (1.71; 1.76)	2.98 (2.90; 3.06)	4.18 (4.09; 4.27)	2.10 (2.07; 2.14)	1.63 (1.61; 1.65)
Age 80–89	1.15 (1.13; 1.17)	1.49 (1.46; 1.53)	2.75 (2.66; 2.85)	3.23 (3.13; 3.33)	1.83 (1.79; 1.86)	1.48 (1.46; 1.51)
Age > = 90	1.03 (0.98; 1.08)	1.30 (1.21; 1.39)	2.50 (2.24; 2.79)	2.75 (2.47; 3.05)	1.60 (1.49; 1.72)	1.28 (1.21; 1.35)
Interaction population*chronic conditions ^ⱡ^	*p* < 0.0001	*p* < 0.0001	*p* < 0.0001	*p* < 0.0001	*p* < 0.0001	*p* < 0.0001
0 chronic condition	1.50 (1.49; 1.52)	2.23 (2.20; 2.26)	4.35 (4.26; 4.44)	5.84 (5.74; 5.94)	2.63 (2.60; 2.67)	2.03 (2.01; 2.05)
1 chronic condition	1.32 (1.30; 1.33)	1.74 (1.72; 1.77)	3.04 (2.98; 3.11)	4.14 (4.06; 4.21)	2.07 (2.05; 2.10)	1.64 (1.62; 1.66)
2 chronic conditions	1.24 (1.22; 1.26)	1.51 (1.48; 1.54)	2.59 (2.50; 2.67)	3.19 (3.10; 3.28)	1.80 (1.77; 1.84)	1.50 (1.47; 1.52)
>2 chronic conditions	1.22 (1.19; 1.26)	1.45 (1.40; 1.50)	2.29 (2.16; 2.41)	2.86 (2.72; 3.01)	1.72 (1.66; 1.79)	1.50 (1.45; 1.54)

The control group is used as the reference population. With respect to the control population: The complete year before hospital admission is used for the analyses with respect to the year before hospital admission, since Fig 2 showed no differences in mean number of general practitioner consultations during the year before hospital admission for the control group. The complete year after hospital discharge is used for the analyses with respect to the year after hospital discharge, since Fig 2 showed no differences in mean number of general practitioner consultations during the year after hospital discharge for the control group.

‡ Corrected for SES age and chronic conditions

¥ Corrected for gender, age and chronic conditions

† Corrected for gender SES and chronic conditions

ⱡ Corrected for gender, SES and age

CI: Confidence Interval; ICU: Intensive Care Unit; SES: Socioeconomic status

Women had 1.36 (CI 1.35; 1.37) times more GP consultations compared to men during period 1 (Table 3). During period 3 the difference between men and women in the ICU population was smaller (RR 1.22 (CI 1.19; 1.25) but still present. Women in the ICU population had 1.07 (CI 1.04; 1.09) more consultations compared to men during period 3 and 1.29 (CI 1.28; 1.30) more consultations during period 6.

**Table 3:** Risk Ratios (95% CI) of GP consultations for different risk factors for the ICU population and the control group separately.

	Year before hospital admission				Year after hospital discharge			
	Control group week 52–1	ICU population Period 1 (week 52–17)	ICU population Period 2 (week 16–5)	ICU population Period 3 (weeks 4–1)	Control group week 1–52	ICU population Period 4 (week 1–4)	ICU population Period 5 (week 5–16)	ICU population Period 6 (week 17–52)
Male (reference)	1	1	1	1	1	1	1	1
Female^‡^	1.33 (1.32; 1.34)	1.36 (1.35; 1.37)	1.26 (1.24; 1.28)	1.22 (1.19; 1.25)	1.31 (1.29; 1.33)	1.07 (1.04; 1.09)	1.21 (1.19; 1.22)	1.29 (1.28; 1.30)
SES Q1 (reference)	1	1	1	1	1	1	1	1
SES Q2^¥^	0.96 (0.95; 0.98)	0.99 (0.98; 1.00)	1.01 (0.99; 1.03)	1.01 (0.97; 1.04)	0.96 (0.95; 0.98)	1.07 (1.04; 1.10)	1.01 (0.99; 1.03)	1.01 (1.00; 1.02)
SES Q3^¥^	0.96 (0.95; 0.98)	0.98 (0.97; 0.99)	1.00 (0.98; 1.02)	1.02 (0.99; 1.06)	0.94 (0.92; 0.96)	1.08 (1.05; 1.11)	1.01 (0.99; 1.03)	1.00 (0.98; 1.01)
SES Q4^¥^	0.92 (0.91; 0.94)	0.94 (0.93; 0.96)	1.00 (0.98; 1.02)	0.99 (0.96; 1.03)	0.91 (0.90; 0.93)	1.08 (1.04; 1.11)	0.99 (0.97; 1.01)	0.97 (0.96; 0.98)
Age 18–29 (reference)	1	1	1	1	1	1	1	1
Age 30–39^†^	1.05 (1.01; 1.10)	1.04 (1.00; 1.08)	1.12 (1.06; 1.19)	1.13 (1.02; 1.25)	1.06 (1.00; 1.12)	1.17 (1.08; 1.28)	1.16 (1.09; 1.22)	1.09 (1.05; 1.13)
Age 40–49^†^	1.12 (1.08; 1.16)	1.07 (1.03; 1.10)	1.20 (1.14; 1.26)	1.28 (1.18; 1.40)	1.13 (1.08; 1.18)	1.29 (1.20; 1.39)	1.30 (1.24; 1.37)	1.21 (1.18; 1.25)
Age 50–59^†^	1.21 (1.17; 1.25)	1.06 (1.03; 1.09)	1.22 (1.17; 1.28)	1.22 (1.12; 1.32)	1.22 (1.17; 1.27)	1.32 (1.23; 1.41)	1.35 (1.29; 1.42)	1.29 (1.26; 1.33)
Age 60–69^†^	1.37 (1.32; 1.41)	1.04 (1.01; 1.07)	1.29 (1.23; 1.35)	1.26 (1.17; 1.37)	1.43 (1.37; 1.49)	1.45 (1.35; 1.54)	1.46 (1.40; 1.53)	1.33 (1.30; 1.37)
Age 70–79^†^	1.76 (1.70; 1.81)	1.18 (1.15; 1.21)	1.46 (1.39; 1.52)	1.37 (1.27; 1.48)	1.87 (1.79; 1.95)	1.57 (1.47; 1.68)	1.70 (1.62; 1.77)	1.52 (1.48; 1.56)
Age 80–89^†^	2.28 (2.21; 2.36)	1.41 (1.37; 1.45)	1.64 (1.57; 1.72)	1.65 (1.53; 1.79)	2.49 (2.39; 2.60)	1.62 (1.51; 1.73)	1.95 (1.87; 2.05)	1.83 (1.77; 1.88)
Age > = 90^†^	3.06 (2.92; 3.20)	1.69 (1.61; 1.77)	1.92 (1.78; 2.06)	2.02 (1.78; 2.28)	3.29 (3.09; 3.50)	1.82 (1.62; 2.03)	2.25 (2.09; 2.43)	2.07 (1.97; 2.17)
0 chronic condition (reference)	1	1	1	1	1	1	1	1
1 chronic condition^ⱡ^	1.59 (1.58; 1.61)	1.40 (1.38; 1.41)	1.21 (1.19; 1.23)	1.08 (1.05; 1.11)	1.48 (1.46; 1.50)	1.05 (1.03; 1.07)	1.16 (1.14; 1.18)	1.21 (1.20; 1.23)
2 chronic conditions^ⱡ^	2.16 (2.13; 2.19)	1.79 (1.76; 1.81)	1.41 (1.39; 1.44)	1.23 (1.19; 1.27)	1.99 (1.95; 2.03)	1.09 (1.06; 1.12)	1.36 (1.33; 1.38)	1.49 (1.48; 1.51)
>2 chronic conditions^ⱡ^	2.84 (2.76; 2.91)	2.31 (2.27; 2.35)	1.78 (1.74; 1.83)	1.42 (1.36; 1.49)	2.51 (2.43; 2.60)	1.23 (1.18; 1.28)	1.63 (1.59; 1.68)	1.88 (1.85; 1.92)

With respect to the control population: The complete year before hospital admission is used for the analyses with respect to the year before hospital admission, since Fig 2 showed no differences in mean number of general practitioner consultations during the year before hospital admission for the control group. The complete year after hospital discharge is used for the analyses with respect to the year after hospital discharge, since Fig 2 showed no differences in mean number of general practitioner consultations during the year after hospital discharge for the control group.

^‡^ Corrected for SES age and chronic conditions

^¥^ Corrected for gender, age and chronic conditions

† Corrected for gender SES and chronic conditions

ⱡ Corrected for gender, SES and age

CI: Confidence Interval; ICU: Intensive Care Unit; SES: Socioeconomic status

ICU patients of all SES quartiles had more GP consultations compared to individuals from the control group during the year before hospital admission, these differences increased over time. For ICU patients with the highest SES quartile the difference increased most. During the year after hospital discharge a reversed trend was observed; ICU patients of all SES quartiles had more GP consultations compared to the control group but these differences decreased over time.

Within the ICU population, ICU patients from the lowest SES quartile had 1.12 (CI 1.09; 1.16) times more GP consultations compared to ICU patients from the highest SES quartile. During period 3 this difference was 1.06 (CI 1.00; 1.13) (Table 3).

The difference in GP consultations between the ICU population and the control group with respect to age was most distinct within the younger age groups and this difference increased closer to the time of hospital admission. Within the ICU population, older patients had more GP consultations compared to younger patients. The differences between age groups within the ICU population became smaller closer to the time of admission.

ICU patients with an elective surgical admission or an emergency surgery admission had less consultations with the GP compared to ICU patients with a medical admission during period 1 with a RR of respectively 0.86 (CI 0.85; 0.87) and 0.83 (CI 0.82; 0.84). During period 4, ICU patients with an elective surgical admission or an emergency surgery had more consultations with the GP compared to ICU patients with a medical admission (RR 1.05 (CI 1.00; 1.11) and RR 1.08 (CI 1.00; 1.17) respectively) (Table 4).

**Table 4:** Risk Ratios (95% CI) for GP consultations for different risk factors among the ICU population.

	Year before hospital admission			Year after hospital discharge		
	Period 1 (week 52–17)	Period 2 (week 16–5)	Period 3 (weeks 4–1)	Period 4 (weeks 1–4)	Period 5 (weeks 5–16)	Period 6 (weeks 17–52)
Admission type						
Medical (reference)	1	1	1	1	1	1
Emergency surgery	0.83 (0.82; 0.84)	0.87 (0.85; 0.89)	0.94 (0.84; 1.06)	1.08 (1.00; 1.17)	0.98 (0.96; 1.01)	0.90 (0.89; 0.91)
Elective surgery	0.86 (0.85; 0.87)	1.01 (0.99; 1.02)	0.65 (0.59; 0.70)	1.05 (1.00; 1.11)	0.88 (0.86; 0.89)	0.86 (0.85; 0.87)
Length of ICU stay						
< 2 days (reference)	1	1	1	1	1	1
> = 2 days < 5 days	1.03 (1.02; 1.05)	0.98 (0.96; 1.00)	1.21 (1.11; 1.32)	1.06 (1.00; 1.13)	1.09 (1.07; 1.11)	1.09 (1.08; 1.10)
> = 5 days	0.99 (0.97; 1.00)	0.92 (0.90; 0.94)	1.21 (1.09; 1.35)	0.97 (0.90; 1.05)	1.12 (1.10; 1.15)	1.14 (1.13; 1.16)
APACHE IV risk group						
Low (reference)	1	1	1	1	1	1
Medium	1.05 (1.03; 1.06)	1.03 (1.01; 1.06)	1.33 (1.19; 1.48)	1.02 (0.93; 1.11)	1.16 (1.13; 1.19)	1.14 (1.13; 1.16)
High	0.90 (0.87; 0.92)	0.88 (0.84; 0.93)	1.07 (0.86; 1.32)	0.91 (0.78; 1.07)	1.05 (1.00; 1.11)	1.01 (0.98; 1.04)

APACHE IV: Acute Physiology and Chronic Health Evaluation IV; CI: Confidence Interval; ICU: Intensive Care Unit

ICU patients with an ICU length of stay of 2 to 5 days had more consultations with a GP compared to ICU patients with a length of stay <2 days during the year before admission and the year after discharge and ICU patients with a medium risk of mortality had more GP consultations compared to ICU patients with a low risk of mortality during the year before admission and the year after discharge (Table 4).

## Discussion

This study showed that ICU patients have more consultations with GPs during the year before and the year after hospital admission compared to a matched control group. Shortly before hospital admission and shortly after hospital discharge, the number of GP contacts is substantially increased. During the last four weeks before admission, ICU patients have 3.58 (CI 3.37; 3.80) times more GP consultations compared to the control group. During the first four weeks after discharge, ICU patients have 4.98 (CI 4.74; 5.23) times more GP consultations. One year after hospital discharge, ICU patients have 1.91 (CI 1.88; 1.93) times more GP consultations compared to the control group; this is still higher than the same period before hospital admission (RR 1.55 (CI 1.53; 1.57).

During period 1 (52 to 17 weeks before hospital admission), ICU survivors already had more GP consultations compared to the control group. Gender, age, SES and multi morbidity are important risk factors for an increased number of GP consultations [21, 22]. Since we matched our two study populations 1:1 on age, gender and SES, and the number of chronic conditions was taken into account as a confounder within the Poisson regression, the difference can only be explained by another factor. We have reason to believe that ICU survivors have an impaired health status and therefore a decreased quality of life long before ICU admission. A systematic review reported that pre-ICU quality of life is low compared to that of the general population, indicating that ICU patients differ from the average population even before the onset of critical illness [23]. Other studies have reported that ICU survivors have a higher healthcare consumption preceding their ICU admission [20, 24] which can be an indication that they have a reduced quality of life long before ICU admission.

During the last four weeks before hospital admission (period 3), the number of GP consultations in the ICU population increased substantially. Other studies reported that prior to their ICU admission, ICU patients have an increased healthcare consumption [20, 24]. Possible explanations for the higher GP consultation rate during this period can be that people experience more health problems and contact a GP. Subsequently the GP can refer the patient to the hospital immediately, or the GP refers the patients to the hospital after a regular check-up, or the GP performs check-ups before elective surgery.

Possible explanations for the high GP consultation rate during the first four weeks after hospital discharge (period 4) are that the GPs contacts the patients since the GP received a discharge letter from the hospital, or the patient contacts the GP because they experience health problems or they need a referral for another healthcare provider. Post-hoc analyses showed that the RR between the ICU population and the control group without all telephone contacts is a only little lower (RR 4.11 (CI 3.92; 4.30)) during the first four weeks after discharge. Thus, the standard healthcare process (a phone call after hospital discharge) does not fully explain the difference in GP consult rate after discharge between ICU patients and the control group. Therefore, we hypothesize that ICU patients contact the GPs more often since they experience more health problems shortly after discharge.

In the period 17 weeks to 52 weeks after discharge (period 6), ICU patients had on average more GP consultations compared to the same weeks before hospital admission (period 1). Previously published studies reported similar findings [25, 26]. ICU patients have a five times higher risk of developing new chronic condition after ICU admission compared to a population-based control group, and they suffer long-term complaints after ICU discharge [17]. People with more chronic conditions have higher healthcare consumption and more GP consultations [20, 27]. This could partly be an explanation for the increased number of GP consultations compared to their situation before ICU admission.

ICU follow-up care has been suggested as a potential means to address the physical, cognitive and mental problems faced after discharge, but it is unknown which (combination of) interventions are most (cost)effective [7, 8]. Studies proposed that frequent visits to GPs allowed early recognition and proactive treatment of health problems that prevented further hospitalizations [28] and that the post-hospital collaboration among hospital, GP and community services regarding physical and neuropsychological rehabilitation should be strengthened [26]. A multi-centre study conducted in the Netherlands reported that, at three months after discharge, almost 70% of the ICU survivors had had contact with the GP. However, half of the ICU survivors with complaints had no contact with the appropriate health professional (other than the GP)[10]. In light of these findings, we suggest that GPs should be informed about the problems ICU patients can suffer after discharge so, if needed, the GP can refer the patient to the appropriate healthcare professional. Future research is necessary to gain insight into the GP’s potential role in organizing care tailored to the needs of ICU survivors.

A limitation of using claims data is that we have no insight into the purpose of the contact between ICU patients and the GP, and that we do not know who initiated the contact. Further research about the purpose of GP consultations and who initiated them could give more insight into the healthcare trajectory of ICU survivors after discharge. Another limitation of using claims data is that we do not have information about the GP consultations of people living in a nursing home. ICU patients have higher healthcare consumption and more chronic conditions before ICU admission and therefore it is possible that patients of the ICU population are more likely to live in a nursing home. This can lead to an underestimation of the true GP consultation rate, especially within the elderly ICU population.

Another limitation is the exclusion of all GP consultations that took place as part of a 'multidisciplinary care arrangement' with the insurer. Since more ICU patients had 'multidisciplinary care arrangements' than the control group we expect that the differences in GP consultations between the ICU population and the control group are slightly larger than we reported in this study. Despite these limitations, we still believe the differences we found are clinically relevant. A strength of this study is that all the data we used for this study was routinely-collected data instead of self-reported data. Moreover, we were able to include almost all patients admitted to a Dutch ICU through the unique collaboration of a national health insurance claims database and a national clinical ICU quality database. This diminishes the risk of selection bias. Since we included almost all ICU patients of an entire country, we believe that the results we found are representative for other Western European countries with similar healthcare systems as well.

## Conclusion

This study showed that ICU patients have more consultations with GPs during the year before and the year after hospital admission compared to a matched control group. Near the time of hospital admission and shortly after hospital discharge, the number of GP contacts substantially increases within the ICU population. We suggest that GPs should be informed about the problems ICU patients suffer after discharge, in order to provide the care they need. More research about how the care delivered by GPs can be integrated in ICU follow-up care is necessary, and is likely to might be beneficial for this large group of patients.

### Ethics

The Medical Ethics Committee of the Academic Medical Center waived the need for ethical approval for this study. Their response is stored under number W18_043.

## Supporting information

S1 Table Number of participants, number of GP consultations, follow-up time and crude Risk Ratio of GP consultations among ICU patients compared to the control group for the different time periods. (PDF)

## Abbreviations

APACHE IV: Acute Physiology and Chronic Health Evaluation IV
CI: Confidence Interval
COPD: Chronic Obstructive Pulmonary Disease
DM: Diabetes Mellitus
GP: General practitioner
ICU: Intensive Care Unit
PICS: Post-intensive Care Syndrome
RR: Risk Ratio
SES: Socioeconomic status
