# Supplementary material for: Dutch ICU survivors have more consultations with general practitioners before and after ICU admission compared to a matched control group from the general population
Source: PLoS One. 2019 May 23;14(5):e0217225. doi: 10.1371/journal.pone.0217225 (PMC6532903; doi:10.1371/journal.pone.0217225)
Supplement: S1 Table — (PDF) [file pone.0217225.s001.pdf]

S1 Table. Number of participants, number of GP consultations, follow-up time and crude Risk Ratio of GP consultations among ICU patients compared to the control group for the different time periods

Period	Control group (n)	GP consultations (n)	Follow-up time (weeks)	ICU population (n)	GP consultations (n)	Follow-up time (weeks)	Risk Ratio (95% CI)
During the year before hospital admission	56,267	326,893	2,916,176	56,267	597,307	2,925,884	1.82 (1.80; 1.85)
1 (week 52-17)	56,267	225,086	2,022,398	56,267	349,410	2,025,612	1.55 (1.53; 1.57)
2 (week 16-5)	55,989	76,017	670,772	56,267	155,020	675,204	2.03 (1.98; 2.08)
3 (week 4-1)	55,774	25,790	223,006	56,267	92,877	225,068	3.58 (3.37; 3.80)
During the year after hospital discharge	55,719	326,380	2,868,684	56,267	707,305	2,733,090	2.28 (2.24; 2.31)
4 (week 1-4)	55,719	24,890	222,726	56,267	123,441	222,414	4.98 (4.74; 5.23)
5 (week 5-16)	55,617	75,081	665,947	54,872	178,058	648,892	2.43 (2.38; 2.49)
6 (week 17-52)	55,340	226,409	1,980,011	53,282	405,806	1,861,784	1.91 (1.88; 1.93)
Total study period	56,267	653273	5,784,860	56,267	1,304,612	5,658,974	2.04 (2.02; 2.06)

CI: Confidence Interval; GP: General practitioner; ICU: Intensive Care Unit
